# Design and Production of Continuously Gradient Macro/Microporous Calcium Phosphate (CaP) Scaffolds Using Ceramic/Camphene-Based 3D Extrusion

**DOI:** 10.3390/ma10070719

**Published:** 2017-06-28

**Authors:** Min-Kyung Ahn, Young-Wook Moon, Woo-Youl Maeng, Young-Hag Koh, Hyoun-Ee Kim

**Affiliations:** 1 Department of Biomedical Engineering, Korea University, Seoul 136-701, Korea; alsrud4286@hanmail.net (M.-K.A.); godwook@korea.ac.kr (Y.-W.M.), abcd2165@korea.ac.kr (W.-Y.M.); 2 Department of Materials Science and Engineering, Seoul National University, Seoul 151-742, Korea; kimhe@snu.ac.kr

**Keywords:** additive manufacturing, extrusion, porous ceramics, biomedical applications

## Abstract

This study proposes a new type of calcium phosphate (CaP) scaffolds with a continuously gradient macro/microporous structure using the ceramic/camphene-based 3D extrusion process. Green filaments with a continuously gradient core/shell structure were successfully produced by extruding a bilayered feedrod comprised of a CaP/camphene mixture lower part and a pure camphene upper part. The extruded filaments were then deposited in a controlled manner to construct triangular prisms, followed by the assembly process for the production of CaP scaffolds with a gradient core/shell structure. In addition, a gradient microporous structure was created by heat-treating the green body at 43 °C to induce the overgrowth of camphene dendrites in the CaP/camphene walls. The produced CaP scaffold showed a highly macroporous structure within its inner core, where the size of macrochannels increased gradually with an increase in the distance from the outer shell, while relatively larger micropores were created in the outer shell.

## 1. Introduction

Biocompatible, biodegradable ceramics with an open porous structure have been used widely as bone scaffolds for the repair of diseased and damaged bone tissues [1]. These porous scaffolds can provide the 3-dimensional space with biocompatible surfaces, which can facilitate the attachment, proliferation, and differentiation of bone cells, thus leading to fast new bone formation in vivo [2]. Particularly, as a scaffolding material, calcium phosphate (CaP) ceramics can have the biomimetic bone regeneration ability owing to their chemical compositions and crystalline structures similar to those of the inorganic phase of natural bones and teeth [2,3]. However, when formulated into porous structures, these materials have relatively low mechanical strengths compared to those of natural bones and would not effectively withstand loads applied to bone defects [4,5]. In addition, their mechanical properties severely decrease with increasing porosity, which has been one of the biggest obstacles for the production of porous ceramic scaffolds with biomimetic mechanical and biological functions.

Therefore, considerable effort has been made to mimic the clever architecture of natural bones on the macro/micro/nanoscales, in order to achieve both high mechanical properties and excellent bone regeneration ability [5]. One of the most promising approaches to this is to create aligned pore structures, since they can more effectively endure applied compressive loads compared to random porous ceramics [5,6]. Such aligned porous structures can be created using unidirectional freeze-casting techniques, where freezing vehicles (e.g., water, camphene) in ceramic slurries can be unidirectionally solidified as dendrites along the direction of heat flow, which can be subsequently removed by freeze-drying [6,7,8,9,10]. In addition, the extrusion of frozen ceramic/camphene bodies can create aligned pores by removing the camphene dendrites extensively elongated through the extrusion process [11,12]. These techniques are very useful in producing aligned porous ceramics with high mechanical strengths; however, they have a limited ability to arbitrarily tailor the local porous structure of porous ceramics.

Another promising approach is to create gradient porous structures, whose motivation is to resemble the spatial distribution of porosity and pore size of natural bones [13,14,15,16,17,18]. For example, bioinspired porous ceramic scaffolds comprised of a dense outer shell and a porous inner core can induce excellent bone ingrowth into the porous core, while providing high mechanical strength due to the dense shell [14,19,20]. In addition, the mechanical and biological functions can be further tailored and optimized for specific bone defects by creating different porous structures at various positions. More recently, additive manufacturing (AM) techniques have demonstrated great potential to more closely mimic the architecture of natural bones, since they can create arbitrarily tailored porous structures (e.g., porosity, pore size, and pore configuration) according to predetermined computer-aided design (CAD) files [21,22,23,24,25,26,27]. Thus, porous ceramic scaffolds produced using these AM techniques can have both high mechanical properties [28,29,30,31,32,33,34,35] and excellent bone regeneration ability [36], and their functions can be further tailored to specific bone defects [37,38].

**Figure 1:**
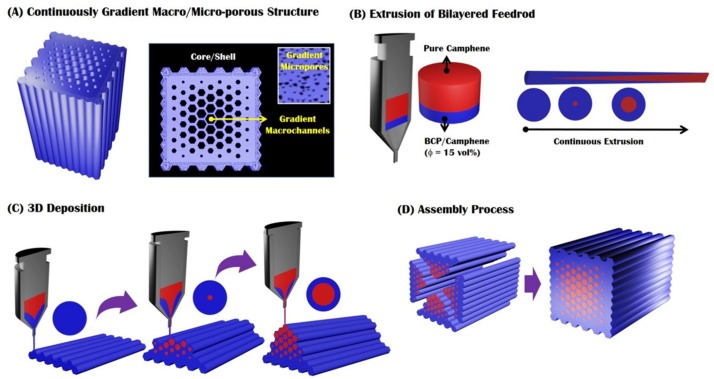
Schematic diagrams of the 3D extrusion of a bilayered feedrod for the production of bioinspired CaP scaffolds with a continuously gradient macro/microporous structure: (**A**) the bioinspired CaP scaffold with a continuously gradient macro/microporous structure; (**B**) the extrusion of a bilayered feedrod, comprised of a CaP/camphene body with a CaP content of 15 vol % and a pure camphene as the lower and upper parts, respectively, for the creation of green filaments with a continuously gradient core/shell structure; (**C**) 3D deposition of the extruded filaments for producing a triangular prism with a unique gradient macrostructure; and (**D**) assembly process and subsequent heat-treatment to produce bioinspired porous CaP scaffolds with a continuously gradient macro/microporous structure.

In this study, we propose a new type of bioinspired CaP scaffold with a continuously gradient macro/microporous structure and a method for its production using the ceramic/camphene-based freeze-casting, as shown in Figure 1A–D. This unique gradient structure can more closely resemble the hierarchical architecture of natural bones with a porous core/dense shell structure. More specifically, the scaffold is comprised of a macroporous inner part having various macrochannels with a gradual increase in their size from an outer part having elongated micropores (Figure 1A). For this goal, we employ a ceramic/camphene-based feedstock with a bilayered structure for 3D extrusion—a frozen ceramic/camphene mixture as the lower part and pure camphene as the upper part. As extrusion proceeds, the pure camphene at the center can be extruded much faster than the ceramic/camphene mixture owing to the wall slip phenomena [39,40,41]. This can allow for the production of green filaments comprised of a pure camphene core and a ceramic/camphene shell, while the core/shell thickness ratio increases gradually (Figure 1B). The extruded filaments can be deposited layer-by-layer in a controlled manner to produce a triangular prism, where the fraction of the camphene core surrounded by the ceramic/camphene shell can increase gradually from the bottom to the top (Figure 1C). Subsequently, four triangular prisms are assembled into a single body by gently pressing them into a mold owing to the excellent adhesive nature of the ceramic/camphene body [34,35] (Figure 1D). To create a gradient microporous structure, the green body is heat-treated at 43 °C, which would induce the overgrowth of the camphene dendrites formed in the CaP/camphene parts. After which, the camphene core and dendrites formed in the ceramic/camphene walls are removed by freeze-drying, thus resulting in the formation of a continuously gradient macroporous structure with various macrochannel sizes, as well as elongated micropores in the CaP walls (cf. Figure 1A). Porous CaP ceramic scaffolds with a bioinspired gradient porous structure were produced using the present approach, and their macro/micro-structure was characterized.

## 2. Results and Discussion

### 2.1. 3D Extrusion and Assembly Process for Gradient Macrostructure

**Figure 2:**
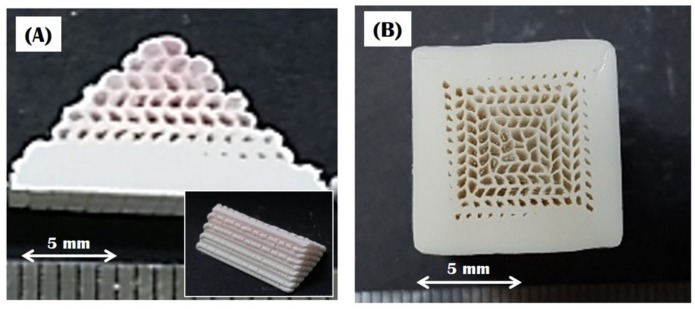
(**A**) Optical image of the green triangular prism showing a gradual change in macrostructure and (**B**) optical image of the assembled body after freeze-drying showing a continuously gradient macrostructure, comprised of the inner core and outer shell. The camphene parts appear red for clarification.

A triangular prism having a gradual increase in the fraction and size of the camphene cores from the bottom was successfully produced using the 3D extrusion of a bilayered feedrod, as shown in Figure 2A. The bottom made of a CaP/camphene mixture was formed at the early stage of extrusion. However, beyond this region, camphene cores began to appear, and their size increased gradually from the bottom to the top. Such a unique gradient macrostructure could be achieved via the preferential extrusion of pure camphene used as the upper part owing to the wall slip phenomena [39,40,41] (cf. Figure 1B). It should be noted that the gradient macrostructure of the green sample would be affected not only by the height of the lower and upper parts, but also by several processing parameters, such as extrusion velocity, shear rate, and working temperature [41]. A green body with a unique gradient macrostructure was successfully produced by assembling four triangular prisms in a rigid die at room temperature. Particularly, to induce the overgrowth of camphene dendrites formed in the CaP/camphene walls, the green body was heat-treated at 43 °C close to the solidification point of a CaP/camphene slurry [12]. It should be noted that such a heat-treatment would not cause noticeable changes in the shape and dimensions of the sample, since it can be carried out at a temperature below the melting point of the CaP/camphene body. Figure 2B shows a representative optical image of the sample after freeze-drying. The sample showed negligible shrinkage, since the frozen camphene dendrites could be removed via sublimation without altering the structure of the CaP walls. Good bonding between the triangular prisms was obtained owing to the excellent adhesive characteristic of the frozen ceramic/camphene body. In addition, the sample preserved the original gradient macrostructure without noticeable distortion. This finding suggests that the simple assembly of extruded ceramic/camphene bodies and post-treatment at 43 °C can be very useful to produce bioinspired CaP scaffolds with a unique continuously gradient macrostructure.

### 2.2. 3D Structure of Bioinspired Gradient Porous CaP Scaffolds

**Figure 3:**
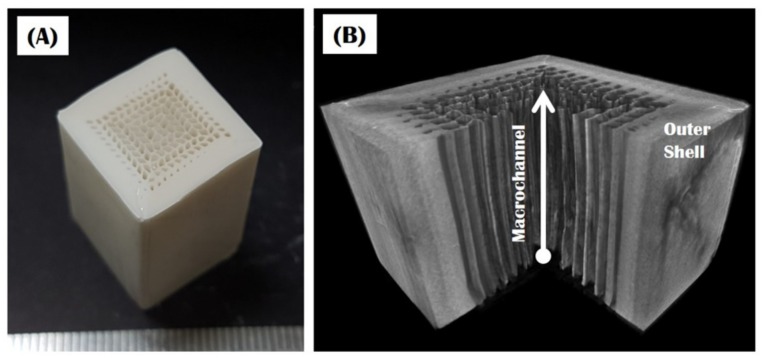
(**A**) Optical image and (**B**) reconstructed μ-CT image of the bioinspired CaP scaffold, showing the external and internal macroporous structures.

Bioinspired porous CaP scaffolds with a unique gradient core/shell structure were produced after sintering at 1250 °C for 2 h. The sample showed good shape tolerance without noticeable defects, such as cracks and delamination at interfaces between the core and shell, as shown in Figure 3A. A uniform linear shrinkage of ~16.5% was observed for the sample. This finding suggests that the final dimensions of porous CaP scaffolds can be reliably controlled by designing an initial structure with larger predetermined dimensions. The overall structure and internal macroporous structure of the sample were more closely examined by μ-CT, as shown in Figure 3B. The sample was comprised of an outer shell without noticeable features and a highly porous core containing straight macrochannels of various sizes—a gradual increase in size from the outer shell.

### 2.3. Gradient Macroporous Structure

**Figure 4:**
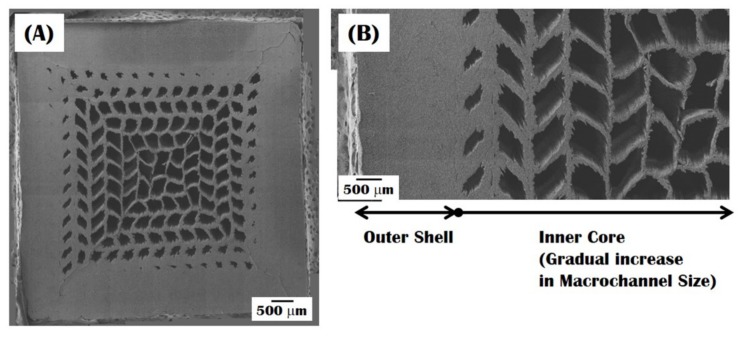
Representative field emission scanning electron microscopy (FE-SEM) image of the bioinspired CaP scaffold showing a gradual change in macroporous structure at (**A**) low and (**B**) high magnifications.

The construction of a gradient macroporous structure was more closely examined by field emission scanning electron microscopy (FE-SEM), as shown in Figure 4A,B. The sample showed a unique gradient macroporous structure, where the macrochannels became larger with an increase in the distance from the dense bottom layer, while the CaP walls became thinner (Figure 4A). In addition, no noticeable defects, such as cracks in the CaP walls or interfacial delamination between the outer shell and inner core, were observed (Figure 4B). This finding suggests that a gradual increase in macroporosity from the outer shell to inner core can effectively reduce shrinkage mismatch during sintering, thus avoiding the interfacial delamination often observed in dense/porous layered composites.

**Figure 5:**
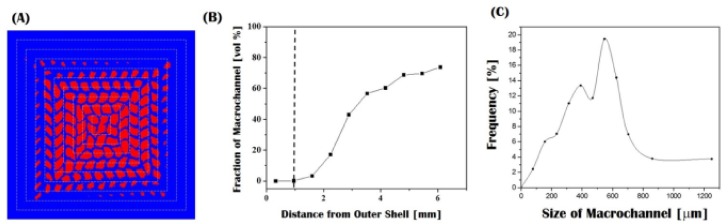
(**A**) Digitally colored image of the sample made for pore size and porosity measurements, in which the macrochannels and CaP walls appear red and blue, respectively; (**B**) fraction of macrochannels as a function of the distance from the outer shell; and (**C**) size distribution of macrochannels computed from the micro- micro-computed (CT) analyses.

The changes in the size and fraction of the macrochannels across the sample were roughly calculated on the basis of SEM images, as shown in Figure 5A,B. For clarification, the macrochannels and CaP walls were digitally colored red and blue, respectively (Figure 5A). The size of the macrochannels increased gradually from ~297 μm to 950 μm in a horizontal direction. In addition, the size distribution of the macrochannels was evaluated using Skyscan CTAn analysis on the basis of micro-CT image (cf. Figure 3B), as shown in Figure 5C. These pore size ranges are expected to provide a favorable environment for bone ingrowth into macrochannels when used as scaffolds for dental and orthopedic applications [1,2]. In addition, the macroporosity (i.e., the fraction of macrochannels), computed by considering the areas of the macrochannels and CaP walls, increased gradually up to ~80 vol % with an increase in the distance from the outer shell (Figure 5B). It should be noted that such a gradient macroporous structure can mimic the structure of natural bones with a relatively dense shell and a highly porous core, thus providing bioinspired functions when used as bone scaffolds.

### 2.4. Gradient Microporous Structure

**Figure 6:**
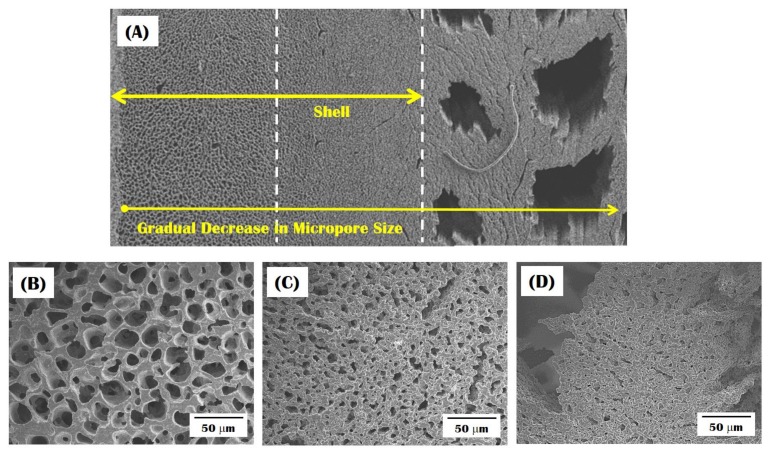
Representative FE-SEM image of the bioinspired CaP scaffold showing a gradual change in microporous structure: (**A**) the overall structure; (**B**) micropores formed in the outer shell; (**C**) micropores formed in the region near the interface between the shell and core; and (**D**) micropores formed in the CaP wall in the macroporous core.

The microporous structures of the CaP walls formed in the shell and core regions were examined by FE-SEM, as shown in Figure 6A–D. The micropores were successfully created as the replica of camphene dendrites formed in the frozen CaP/camphene walls. In addition, interestingly, the sample showed a unique gradient microporous structure, where the size of micropores decreased with an increase in the distance from the outer shell. That is, relatively large pores were formed within the outside of the shell, marked by the dashed line (Figure 6B). Beyond this region, micropores became smaller (Figure 6C), and thus relatively small micropores were created in the highly macroporous core (Figure 6D). The microporosity (i.e., the fraction of micropores), which was measured by considering the volume and weight of the outer shell, was ~75 vol %. Thus, the overall porosity of the sample could increase up to ~81 vol % in the center of the porous core. It should be noted that the micropores would provide microfeatures for the attachment, proliferation, and differentiation of bone cells and paths for the transport of bloods and nutrients, thus leading to excellent bone regeneration when used as bone scaffolds.

**Figure 7:**
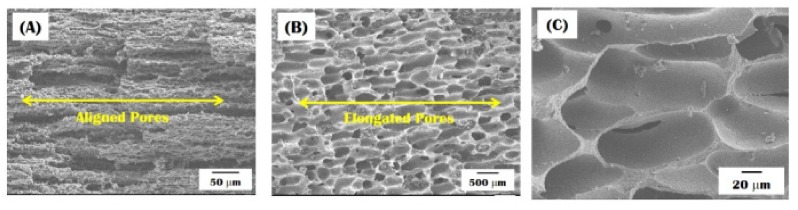
Representative FE-SEM image of the bioinspired CaP scaffold showing (**A**) highly aligned micropores formed in the macroporous core; (**B**) elongated micropores with relatively large sizes formed in the outer shell; and (**C**) the microstructure of the CaP regions.

The microporous structures of the outer shell and inner core along the direction of macrochannels are shown in Figure 7A,B. Highly aligned micropores were formed in the CaP walls within the macroporous core (Figure 7A). On the other hand, the outer shell showed elongated micropores with a significantly larger size compared to the inner core (Figure 7B). In addition, the CaP walls surrounded by the micropores were densified very well without noticeable defects, as shown in Figure 7C. This excellent densification behavior is attributed to the unique phase separation of the CaP/camphene slurry during freeze-casting. More specifically, the fine CaP particles can be ejected by the growing camphene dendrites and then concentrated between the camphene dendrites. These high concentrated ceramic walls (e.g., ceramic content ~50 vol %) can be almost fully densification after sintering at high temperatures, which is one of the most striking advantages of the freeze-casting [11,12].

**Figure 8:**
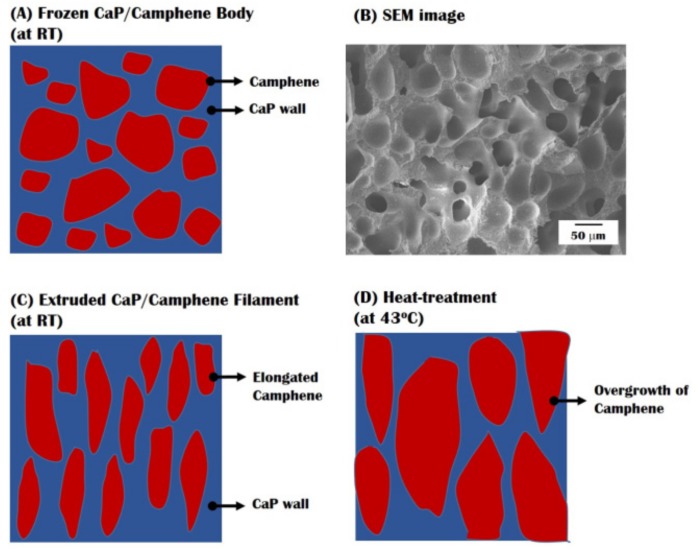
Schematic diagrams showing the change in the morphology of camphene dendrites during various processes: (**A**) freeze-casting at room temperature; and (**B**) corresponding SEM image after the removal of the camphene dendrites; (**C**) extrusion at room temperature; and (**D**) heat-treatment at 43 °C.

It should be noted that the aligned and elongated micropores can be created as replicas of the camphene dendrites that would be extensively elongated through the extrusion process, as is often the case with the extrusion of ceramic/camphene bodies [11,12,30,31], as shown in Figure 8A–D. More specifically, the camphene dendrites can grow randomly without the preferential orientation, the CaP/camphene slurry is frozen at room temperature (Figure 8A). This would result in a uniformly porous structure (Figure 8B). However, the extrusion process can extensively elongate the soft camphene dendrites, thus allowing for the creation of highly elongated pores after the removal of the camphene dendrites (Figure 8C). In addition, the camphene dendrites can grow continuously following its original extensively elongated geometry during heat-treatment, thus resulting in larger micropores with a high aspect ratio (Figure 8D).

To the best of our knowledge, such a gradient microporous structure has not been reported for the ceramic/camphene-based freeze-casting technique even using heat-treatment to induce the overgrowth of camphene dendrites formed in ceramic/camphene walls. That is, the conventional approach would generally result in a relatively uniform porous structure, since camphene dendrites can grow into shapes resembling their original geometry [12]. It was observed that, before heat-treatment at 43 °C, both the outer shell and inner core showed similar micropore sizes (data not shown here). Thus, it is reasonable to suppose that the growth of camphene dendrites during heat-treatment would be strongly affected by the initial gradient macrostructure of the green sample, since the shell and core were exposed at the same temperature of 43 °C. More specifically, the camphene dendrites in the outer shell would grow quite vigorously, resulting in relatively large micropores, as is often the case with the heat-treatment of ceramic/camphene bodies. On the other hand, the growth of the camphene dendrites in the inner core would be hindered, since the CaP/camphene walls are in direct contact with the pure camphene core. Although additional studies are required to interpret this extraordinary phenomenon, it is expected that the size and distribution of micropores could be further tailored by adjusting the dwelling time for heat-treatment.

### 2.5. Water Uptake Capability

**Figure 9:**
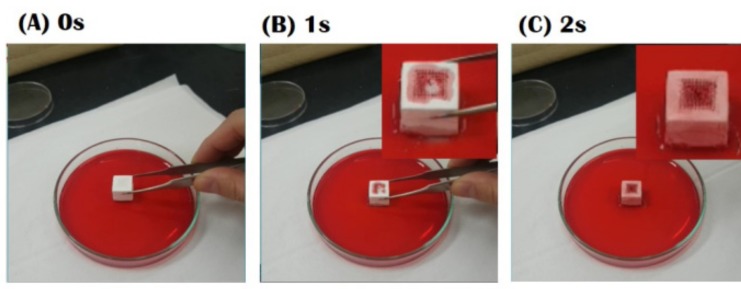
Optical images of the samples in water after various immersion times of (**A**) 0 s; (**B**) 1 s; and (**C**) 2 s. For visualization, a small amount of red dye as added.

The water uptake capability of the CaP scaffold with a bioinspired gradient structure was roughly evaluated by soaking the sample in water, where a small amount of red dye was added for visualization. The entire surfaces of the sample can be completely covered within 2 s, as shown in Figure 9. Such an excellent water uptake capability is attributed to the presence of the 3-dimensionally interconnected micropores, which is one of the most striking features of ceramic/camphene-based freeze-casting. This finding suggests that a CaP scaffold with a bioinspired gradient structure obtained in this study would facilitate the blood flow required for angiogenesis when used as the bone scaffold [1].

### 2.6. Compressive Strength and Stiffness

**Figure 10:**
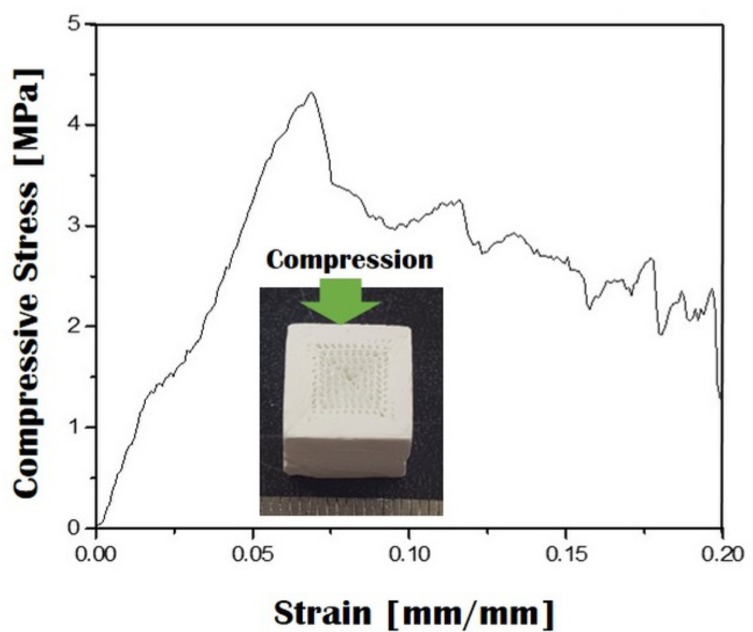
Representative compressive strain versus strain response of the bioinspired CaP scaffold. The sample was compressed to normal to the direction of macrochannels (inset in Figure 10).

The mechanical properties of the CaP scaffold with a bioinspired gradient structure were evaluated using compressive strength tests. The sample was compressed normal to the direction of macrochannels. The typical compressive stress and strain response of the sample is shown in Figure 10. The sample showed a reasonably high compressive strength of 5.23 ± 0.84 MPa and a stiffness of 92.9 ± 12.5 MPa, which would find useful applications as the bone scaffolds [1,2].

## 3. Materials and Methods

### 3.1. Starting Materials

As the starting ceramic powder, commercial calcium phosphate (CaP; OssGen Co., Daegu, Korea) with a mean size of 0.5 μm was used. Commercially available camphene (C_10_H_16_, Sigma Aldrich, St. Louis, MO, USA) with a melting point of ~48–52 °C (manufacturer’s data) was used as the freezing vehicle and fugitive for the creation of micropores and macrochannels, respectively.

### 3.2. CaP/Camphene Slurry Preparation

A CaP/camphene slurry was prepared by mixing the CaP powder and camphene with the assistance of 3 wt % of an oligomeric polyester dispersant (Hypermer KD-4, UniQema, Everburg, Belgium) using ball milling at 60 °C for 24 h. In particular, a relatively low ceramic content of 15 vol % was used to create micropores in the CaP walls.

### 3.3. Bilayered Feedrod Preparation

A rod composed of frozen CaP/camphene mixture was produced by casting the prepared CaP/camphene slurry into a mold with a diameter of 20 mm, followed by freezing at room temperature. In a similar way, a pure camphene rod was prepared using the molten camphene. For visualization, a small amount of red dye was added to the camphene. Subsequently, the CaP/camphene and pure camphene rods were stacked in sequence and then gently pressed to produce a bilayered feedrod for 3D extrusion (cf. Figure 1A). The heights of the lower and upper parts were 5 mm and 13 mm, respectively, which would determine the thickness of the outer and inner parts.

### 3.4. Gradient Porous CaP Scaffold Production Using 3D Extrusion

To produce a triangular prism, the bilayered feedrod was extruded through a reduction die with a diameter of 1 mm at a constant speed of 1 mm/min and then deposited at a travel speed of 6.5 mm/s layer-by-layer in a controlled manner using a computer-controlled moving machine (Jimotor Co., Seoul, Korea) (cf. Figure 1B). The extruded filament had a diameter of ~1 mm with negligible die swell.

After which, four prisms were assembled in a mold and then gently pressed at room temperature, in order to produce a green body with a unique gradient macrostructure (cf. Figure 1C).

**Figure 11:**
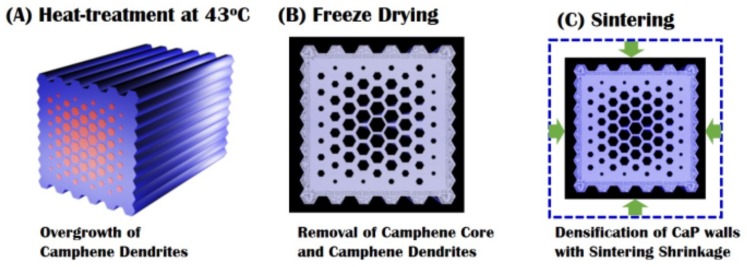
Schematic diagrams showing (**A**) heat-treatment at 43 °C to induce overgrowth the camphene dendrites; (**B**) freeze-drying to remove the camphene core and dendrites; and (**C**) sintering 1250 °C for 2 h for densification of the CaP walls.

The green samples were treated at 43 °C, which would be close to the solidification temperature of the CaP/camphene slurry, to induce the overgrowth of camphene dendrites formed in the CaP/camphene walls (Figure 11A). Subsequently, the green sample was freeze-dried for 24 h to completely remove the camphene used as the core and freezing vehicle (Figure 11B). The samples were then sintered at 1250 °C for 2 h to densify the CaP walls (Figure 11C).

### 3.5. Gradient Macro/Microporous Structure Evaluation

The 3D macro/microporous structure and microstructure of the CaP scaffolds with a bioinspired gradient structure were characterized using several analysis tools. The overall 3D structure and internal macroporous structure were examined by micro-computed tomography (μ-CT, Skyscan 1173 X-ray Micro-tomography System, Skyscan, Kontich, Belgium). For visualization, three-dimensional (3-D) image reconstruction was performed using the NRecon V1.6 program (Skyscan, Kontich, Belgium). In addition, field emission scanning electron microscopy (FE-SEM, JSM-6701F, JEOL Techniques, Tokyo, Japan) was used to more closely examine the macro/microporous structure of the sample and microstructure of the CaP walls. The size and fraction of the macrochannels in the core were roughly measured from the FE-SEM images of the samples. Local porosity was also computed by considering the areas of the macrochannels and CaP walls to demonstrate a gradual change in porosity and pore size toward the center of the sample.

### 3.6. Water-Uptake Capability Evaluation

The water-uptake capability of the CaP scaffold with a bioinspired gradient structure was roughly evaluated by soaking the sample in water. For visualization, as small amount of red dye was added to water.

### 3.7. Compressive Strength Evaluation

The compressive strength of the CaP scaffold with a bioinspired gradient structure was characterized using a screw-driven load frame (OTU-05D, Oriental TM Corp., Seoul, Korea). The samples were uniaxially compressed to normal to the direction of macrochannels. The stress and strain responses of the samples were monitored during the compressive strength tests. Five samples were tested to obtain the mean value and standard deviation.

## 4. Conclusions

Bioinspired CaP scaffolds with a unique, continuously gradient macro/microporous structure were successfully produced by a combination of the 3D extrusion of a bilayered feedrod and subsequent heat-treatment at 43 °C used for the growth of camphene dendrites. The produced CaP scaffold showed a continuously gradient macroporous structure, in which the size of the macrochannels increased gradually from ~290 μm to 930 μm with an increase in distance from the outer shell and thus the macroporosity (i.e., the fraction of macrochannels) increased gradually from 23 vol % to 74 vol %. In addition, the heat-treatment of the sample at 43 °C allowed for the creation of a gradient microporous structure. Highly aligned micropores and elongated micropores with relatively large sizes were formed in the macroporous core and outer shell without noticeable macrofeatures, respectively. Such a unique gradient macro/microporous structure would more closely mimic the architecture of natural bones, thus finding very useful applications in bone tissue engineering.
